# Development and Validation of a Novel Recurrence Risk Stratification for Initial Non-muscle Invasive Bladder Cancer in Asia

**DOI:** 10.1016/j.ebiom.2016.08.051

**Published:** 2016-09-02

**Authors:** Takeshi Ieda, Satoru Muto, Fumitaka Shimizu, Masataka Taguri, Shigeto Yanada, Kousuke Kitamura, Kazutaka Terai, Keisuke Saito, Tatsuya Ogishima, Masayoshi Nagata, Hisamitsu Ide, Takatsugu Okegawa, Yoshiaki Wakumoto, Yoshiro Sakamoto, Akira Tsujimura, Raizo Yamaguchi, Kikuo Nutahara, Shigeo Horie

**Affiliations:** a Department of Urology, Juntendo University, Graduate School of Medicine, Japan; b Department of Urology, Teikyo University, School of Medicine, Japan; c Department of Urology, Juntendo Nerima Hospital, Japan; d Department of Biostatistics, Yokohama City University, School of Medicine, Japan; e Department of Urology, Juntendo Urayasu Hospital, Japan; f Department of Urology, Tokushukai Hospital, Chiba, Japan; g Department of Urology, Kyorin University School of Medicine

**Keywords:** Non-muscle invasive bladder cancer, Recurrence, Risk factor, Classification

## Abstract

**Background:**

Some risk classifications to determine prognosis of patients with non-muscle invasive bladder cancer (NMIBC) have disadvantages in the clinical setting. We investigated whether the EORTC (European Organization for Research and Treatment of Cancer) risk stratification is useful to predict recurrence and progression in Japanese patients with NMIBC. In addition, we developed and validated a novel, and simple risk classification of recurrence.

**Methods:**

The analysis was based on 1085 patients with NMIBC at six hospitals. Excluding recurrent cases, we included 856 patients with initial NMIBC for the analysis. The Kaplan–Meier method with the log-rank test were used to calculate recurrence-free survival (RFS) rate and progression-free survival (PFS) rate according to the EORTC risk classifications. We developed a novel risk classification system for recurrence in NMIBC patients using the independent recurrence prognostic factors based on Cox proportional hazards regression analysis. External validation was done on an external data set of 641 patients from Kyorin University Hospital.

**Findings:**

There were no significant differences in RFS and PFS rates between the groups according to EORTC risk classification. We constructed a novel risk model predicting recurrence that classified patients into three groups using four independent prognostic factors to predict tumour recurrence based on Cox proportional hazards regression analysis. According to the novel recurrence risk classification, there was a significant difference in 5-year RFS rate between the low (68.4%), intermediate (45.8%) and high (33.7%) risk groups (*P* < 0.001).

**Interpretation:**

As the EORTC risk group stratification may not be applicable to Asian patients with NMIBC, our novel classification model can be a simple and useful prognostic tool to stratify recurrence risk in patients with NMIBC.

**Funding:**

None.

## Highlights

• There were no significant differences in RFS & PFS rates between EORTC risk groups.
• We constructed a novel recurrence risk classification using four clinical factors.
• There was a significant difference in RFS rate between the novel risk groups.

Risk classification is essential in any medical treatment. It is constructed based on the large data of observed events such as recurrence and death. However different race/ethnic groups may not follow the prediction since genetic and environmental factors affect the events. Bladder cancer is a frequent cancer among men. We evaluated the commonly-used European risk classification of non-muscle invasive bladder cancer. Surprisingly, this risk classification did not segregate the Japanese patients well. In this study we developed and validated a novel risk model predicting recurrence. This risk classification will be useful among Asian patiens with non-muscle invasive bladder cancer.

## 1 Introduction

Bladder cancer is the fourth most common malignancy in the West (Jemal et al., 2010). In Asia, the incidence of bladder cancer is three to four times less than Western countries (Ferlay et al., 2015). However, population-based cancer registries covering 21% of the world's population only included 8% of patients in these registries are from Asia (Parkin, 2006). At initial diagnosis, about 85% of patients have non-muscle-invasive bladder cancer (NMIBC), which is managed by transurethral resection of the bladder (TUR-Bt) with or without intravesical therapy (Babjuk et al., 2013). Although the prognosis of NMIBC is generally favourable (survival rates at 5 years are above 80%), 50–80% of patients have intravesical recurrence following TUR-Bt (Sylvester et al., 2006, Fernandez-Gomez et al., 2009). Adequate risk classification allows clinicians to not only estimate the clinical behaviour of the tumour, but also the magnitude of benefit and the need for adjuvant therapy. Accordingly, some risk classifications that combine various parameters to estimate the prognosis of NMIBC patients have been reported. There are disadvantages in using these classifications in the clinical setting. For instance, the EORTC(European Organization for Research and Treatment of Cancer) risk table involve complex calculations and imbalance of prevalence of individual risk groups (Sylvester et al., 2006). Furthermore, risk group stratification which takes into account the risk without Bacillus Calmette-Guerin (BCG) instillation or intravesical instillation of chemotherapy has not been reported.

In the present study, we applied the EORTC risk group stratification to predict recurrence and progression in a Japanese cohort. In addition, we developed a novel risk classification of recurrence to easily estimate a NMIBC patient's probability of recurrence after TUR-Bt based on a set of routinely assessed clinical and pathological factors and validated this novel classification using another validation cohort.

## 2 Methods

### 2.1 Inclusion and Exclusion Criteria

In this multicentre retrospective cohort study, we analyzed data from patients with NMIBC who underwent initial TUR-Bt at four Juntendo University Hospitals and Teikyo University Hospital between 2000 and 2013 (JT cohort). To achieve adequate pathological staging, the complete resection aimed to include the muscle layer of the bladder wall. Random biopsies were taken from normal-appearing mucosal area in patients with positive urine cytology and without abnormality of upper and lower urothelial tract. Patients with any of the following were excluded from the analysis: (i) non-urothelial carcinoma histology, (ii) follow-up periods < 3 months: (iii) history of muscle-invasive or metastatic bladder cancer; (iv) history of carcinoma of the urethra, prostate (ducts or stroma), or upper urinary tract; (v) history of local radiation therapy to the pelvis; (vi) history of every kind of chemotherapy; or (vii) history of previous BCG therapy. The TNM classification was assessed based on the 2002 TNM classification of the International Union Against Cancer (Sobin, 2002). The tumour grade was classified according to the World Health Organization system (1973) (Mostofi et al., 1973). This study adhered to the Declaration of Helsinki.

The clinicopathological data, including age, sex, pathological T category, pathological grade, tumour size, number of tumours, presence of concomitant CIS, and intravesical therapy were obtained from each hospital and merged. Each of these variables and their weight (score) adhered to an EORTC scoring system (Sylvester et al., 2006).

Standard cystoscopy and urinary cytological examination, computed tomography with contrast medium if possible, magnetic resonance (MR) imaging and MR urography with contrast medium if possible, were performed every three months for five years after TUR-Bt, and subsequently every six months after five years. No patients had a fluorescence cystoscopy.

### 2.2 Recurrence-Free Survival (RFS), Progression-Free Survival (PFS) and EORTC Risk Classification

Visible recurrences or suspicious lesions were removed by TUR-Bt and biopsy. All recurrences were confirmed by histopathology, and progression was defined as the development of muscle-invasive tumour or metastatic disease. Progression was also regarded as recurrence. Patients without an event were censored at the last date of follow-up. RFS and PFS were defined as the period between the initial TUR-Bt and recurrence or progression, respectively. Patients who died from causes other than urothelial tumour were censored at the time of death.

To evaluate the EORTC risk group stratification for predicting recurrence and progression in JT cohort, a total recurrence score for each patient was calculated based on the six clinicopathological factors according to the EORTC scoring system for recurrence and progression (Babjuk et al., 2008). Patients were then divided into four risk groups for recurrence and progression (Sylvester et al., 2006, Babjuk et al., 2008).

### 2.3 Recurrence Prognostic Factor Analysis and Novel Risk Classification Predicting Recurrence

Univariate and multivariate Cox proportional hazards regression models were used to assess the impact of various clinicopathological factors including age, sex, number of tumours, tumour size, pT, grade, concurrent CIS, BCG instillation, and intravesical instillation of chemotherapy on time to recurrence in JT cohort.

We developed the novel risk classification system for recurrence in NMIBC patients using the independent recurrence prognostic factors based on Cox proportional hazards regression analysis in the JT cohort. Patients were subdivided into low, intermediate and high risk groups according to their total score. Validation was done on an external data set of 641 patients from Kyorin University Hospital. Inclusion and exclusion criteria of the validation set were the same as the JT cohort.

### 2.4 Statistical Analysis

RFS rates were calculated by the Kaplan–Meier method and the difference between each group was evaluated using the log–rank test. Calibration of predictions on the novel risk score was evaluated by comparing the predicted probability at 3 years with the Kaplan–Meier survival probability using the training data (internal calibration). Similar analysis was performed using the external validation data (external calibration). The performance of the predictions was assessed by plotting actual survival against mean of the predicted risks. All statistical analyses were performed using the JMP Pro-11® (SAS Institute Inc., Cary, NC, USA) and SAS version 9.2 (SAS Institute Inc., Cary, NC, USA). *P*-values < 0.05 were considered significant and all reported *P* values were two-sided. This study was conducted in accordance with ethical principles of the Declaration of Helsinki. We registered this study in UMIN clinical trial registry (UMIN000021878).

## 3 Results

### 3.1 Patient Characteristics in JT Cohort (Table 1)

This analysis was based on 1085 patients with NMIBC treated between 2000 and 2013 at the Juntendo and Teikyo University Hospitals. Excluding recurrent cases, there were 856 patients with initial NMIBC (male; *n* = 683, 79.8%, female; *n* = 173, 20.2%) in the JT cohort. The baseline clinical and pathological characteristics of these patients are presented in Table 1. All patients except one were Japanese. The non-Japanese patient was a Caucasian. Median follow-up periods were 31 months (IQR: 15–48). Median age was 71 years old (IQR: 64–78). A 2nd TUR was performed in 134 (15.7%) patients because of T1 or high grade cancer. Based on this, 53 patients (39.6%) were diagnosed with urothelial cancer. Immediate and adjuvant intravesical instillation of chemotherapy were performed in 59 (6.9%) and 56 patients (6.5%), respectively. Two hundred twenty (25.7%) patients were treated by intravesical instillation of BCG. BCG maintenance therapy was performed in only 21 patients (2.5%). According to the EORTC recurrence risk classification, the intermediate risk group had predominantly higher number of patients (751; 87.8%) compared with the low (58; 6.7%) and high risk groups (47; 5.5%). In terms of EORTC progression risk classification, 191 (22.3%) patients were categorized as low-, 341 (39.8%) with intermediate-, and 324 (37.9%) high risk. Radical cystectomy was performed in nine patients (1.1%). Four patients (0.5%) died of bladder cancer and one (0.1%) died of an unrelated disease.

### 3.2 RFS Rates Stratified by the EORTC Recurrence Risk Classification in JT Cohort

**Fig. 1:**
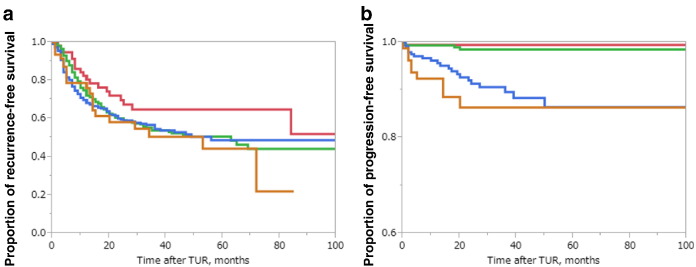
a. Kaplan–Meier RFS curves, stratified by the EORTC recurrence risk classification in JT cohort. There were no significant differences in RFS rate between EORTC risk groups. In the JT cohort, 2-, 3- and 5-year RFS rates of patients for each risk groups were as follows, respectively; low risk: 70.0%, 64.7%, 64.7%, intermediate-low risk: 60.1%, 53.6%, 50.4%, intermediate-high risk; 59.4%, 54.5%, 48.5%, high risk; 58.0%, 50.4%, 44.1%. There were no significant differences in RFS rates between groups according to the EORTC recurrence risk classification (low vs. intermediate-low; *P* = 0.109, intermediate-low vs. intermediate-high; *P* = 0.511, intermediate-high vs. high; *P* = 0.707). red: low risk, green: intermediate-low risk, blue: intermediate-high risk, orange: high risk. b. Kaplan–Meier PFS curves, stratified by the EORTC progression risk classification in JT cohort. Although the differences in PFS rates between patients in intermediate and high-low risk groups were statistically significant (*P* < 0.001), there were no significant difference between low risk group vs intermediate risk (*P* = 0.454), and high-low risk group vs high-high risk group (*P* = 0.338). In the JT cohort, 2-, 3- and 5-year PFS rates of patients for each risk groups were as follows, respectively; low risk: 99.5%, 99.5%, 99.5%, intermediate risk: 98.5%, 98.5%, 98.5%, high-low risk: 91.3%, 89.6%, 86.3%, high-high risk: 86.3%, 86.3%, 86.3%. red: low risk, green: intermediate risk, blue: high-low risk, orange: high-high risk.

During the observation period of this study, 342 of the 856 patients (40.0%) experienced intravesical recurrence. Overall, RFS rates of these patients were 60.3% at 2 years, 54.5% at 3 years, and 50.2% at 5 years. The median time to recurrence was 63.0 months. The RFS rates at 5 years were 64.7% for the low risk group, 50.4% for the intermediate-low risk group, 48.5% for the intermediate-high risk group and 44.1% for high risk group (Fig. 1a). There were no significant differences in RFS rates between groups according to the EORTC recurrence risk classification (low vs. intermediate-low; *P* = 0.109, intermediate-low vs. intermediate-high; *P* = 0.511, intermediate-high vs. high; *P* = 0.707).

### 3.3 PFS Rates Stratified by the EORTC Progression Risk Classification in JT Cohort

Thirty-five (4.1%) patients had disease progression. Overall, PFS rates of the 856 patients were 95.6% at 2 years, 95.0% at 3 years and 94.1% at 5 years. Median PFS rates were incomputable because of the small number of patients with progression. The differences in PFS rates between patients in intermediate and high-low risk group were statistically significant (*P* < 0.001). However, there were no significant difference for the low risk group vs intermediate risk (*P* = 0.454), and high-low risk group vs high-high risk group (*P* = 0.338) (Fig. 1b).

### 3.4 Independent Prognostic Factors to Predict Tumour Recurrence After TUR-Bt in JT Cohort

Univariate and multivariate Cox proportional hazards regression analysis (Table 2) revealed that the number of tumours (*P* < 0.001), tumour size (*P* = 0.003), BCG instillation (*P* < 0.001), and intravesical instillation of chemotherapy (*P* = 0.002) had significant influence on time to recurrence. Other clinical factors including age, sex, pT, grade, concomitant CIS were not statistically significant prognostic factors for recurrence. We could not make an analysis of progression because of the incomputable median PFS.

### 3.5 A Novel Risk Classification Predicting Recurrence After TUR-Bt in the JT Cohort

**Fig. 2:**
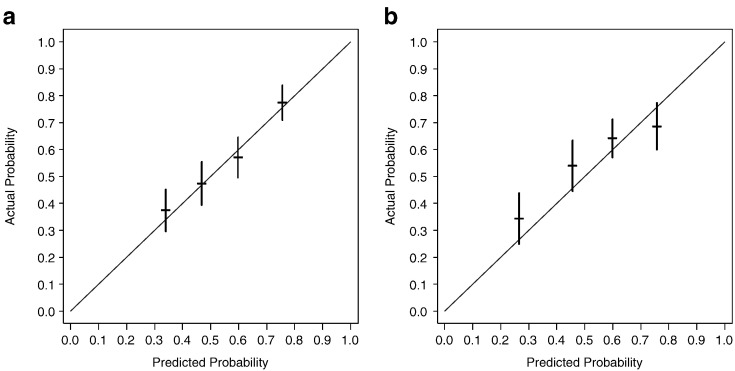
a. Internal validation b. External validation Calibration of risk score for 3-year survival in the internal and external validation data set. The predicted survival rate from the risk score was well correlated with the actual observation. The blue line indicates the ideal reference line where the predicted probabilities match the observed proportions. The dashes represent the nomogram-predicted probabilities grouped for each of the four quartile groups, along with the respective 95% confidence intervals.

We developed a novel risk classification model for recurrence that classified patients into three groups by using weighted scores of clinicopathological factors identified by a univariate Cox proportional hazards regression analysis (number of tumours, tumour size, BCG instillation and intravesical instillation of chemotherapy) in the JT cohort. We showed the 3-year recurrence probability in the JT and validation sets in Table 3. The patients were then divided into three risk groups for recurrence based on their total scores (low risk; total recurrence scores 0–33, intermediate risk; total recurrence scores 34–44, high risk; total recurrence scores 45–57). Calibration of the predictions was evaluated by comparing the predicted probability at 3 years with the Kaplan–Meier survival probability using the training data (internal calibration). The predictions were assessed for calibration accuracy by plotting actual survival against predicted risk (Fig. 2a). The predicted survival rate from the risk score was well correlated with the actual observation of 5-year survival in the training data.

**Fig. 3:**
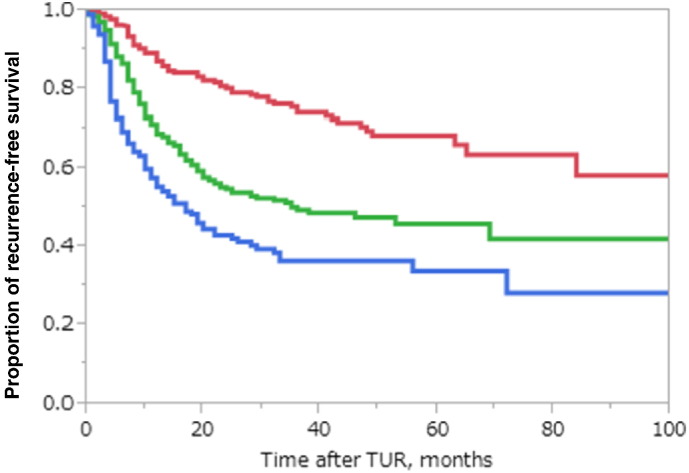
Kaplan–Meier recurrence curves in the JT cohort according to the novel recurrence risk classification There were significant differences in RFS rates between low risk and intermediate risk groups (*P* < 0.001) and between intermediate and high risk groups (*P* < 0.001). The 2-, 3- and 5-year RFS rates of patients for each risk groups were as follows, respectively; low risk: 80.2%, 74.1%, 68.4%, intermediate risk: 54.8%, 49.5%, 45.8%, high risk; 42.1%, 36.3%, 33.7% red: low risk, green: intermediate risk, blue: high risk.

In this novel recurrence risk classification, 280 cases (32.7%) were classified as low risk, 344 (40.1%) as intermediate risk, and 232 (27.1%) as high risk. The RFS rates were 80.2% (at 2 years), 74.1% (at 3 years), 68.4% (at 5 years) for the low risk group; 54.8% (at 2 years), 49.5% (at 3 years), 45.8% (at 5 years) for intermediate risk; 42.1%, (at 2 years), 36.3% (at 3 years), 33.7% (at 5 years) for high risk. There were significant differences in 5-year RFS rates between low risk and intermediate risk (*P* < 0.001) and intermediate risk and high risk (*P* < 0.001) (Fig. 3).

### 3.6 The External Validation of Our Novel Risk Classification Predicting Recurrence After TUR-Bt

We included 641 patients (male; *n* = 501, 78.2%, female; *n* = 140, 21.8%) who were treated at Kyorin University Hospital as external validation cohort. The baseline clinical and pathological characteristics of the validation cohort are presented in Table 1. Although median age (72 years: IQR 62–79, *P* = 0.735) and male-to-female ratio (78.2%, *P* = 0.443) in the validation cohort were similar to the JT cohort, other clinical background including pT (*P* < 0.001), tumour size (*P* = 0.001), number of tumours (*P* < 0.001) and pathological characteristics such as grade (*P* = 0.024) and concomitant CIS (*P* < 0.001) were distinctly different from the JT cohort. In addition, there were fewer 2nd TUR (*P* < 0.001) and BCG induction therapy (*P* < 0.001) in the validation cohort. On the other hand, adjuvant intravesical instillation of chemotherapy (*P* < 0.001) was more frequent in the validation cohort. Radical cystectomy was performed in 43 patients (6.7%). Twenty patients (3.1%) died of cancer and 13 patients (2.0%) died of unrelated disease. Overall, RFS rates of the 641 patients in the validation cohort were 61.1% (at 2 years), 56.2% (at 3 years), and 50.2% (at 5 years). The PFS rates for these patients were 91.7% (at 2 years), 91.0% (at 3 years), 89.1% (at 5 years). Although the PFS rate in the validation cohort was significantly lower than the JT cohort (*P* < 0.001), there were no significant differences in the RFS rate between the groups (*P* = 0.907).

**Fig. 4:**
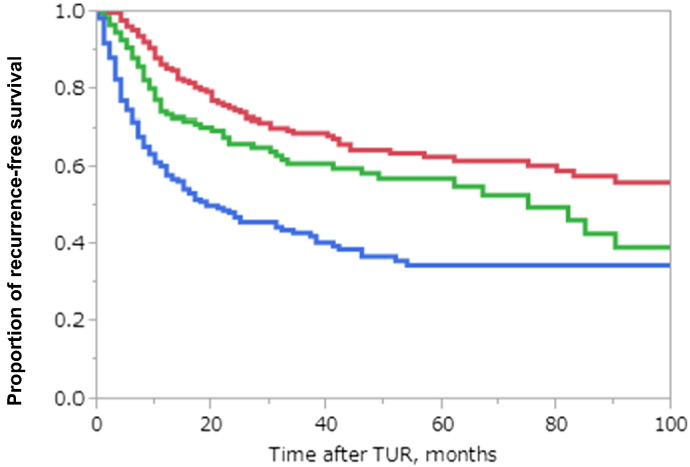
Kaplan–Meier recurrence curves of patients from the validation set according to the novel recurrence classification There were significant differences in RFS rates between low risk and intermediate risk groups (*P* = 0.017) and between intermediate and high risk groups (*P* < 0.001). The 2-, 3- and 5-year RFS rates of patients for each risk groups were as follows, respectively; low risk: 74.5%, 69.0%, 62.9%, intermediate risk: 66.2%, 61.1%, 57.1%, high risk; 47.1%, 43.0%, 34.6% red; low risk, green; intermediate risk, blue; high risk.

According to this novel recurrence-risk classification, 202 cases (31.5%), 159 cases (24.8%) and 280 cases (43.7%) in the validation cohort were classified into low, intermediate, and high risk groups, respectively. There were significant differences in the 5-year RFS rates between the low risk group and intermediate risk group (*P* = 0.017) and between the intermediate risk and high risk groups (*P* < 0.001; Fig. 4).

We also evaluated the calibration by comparing the predicted probability at 3 years with the Kaplan–Meier survival probability using the external validation data (external calibration). Using the validation data set, the predictions were assessed for calibration accuracy by plotting actual survival against predicted risk (Fig. 2b). The predicted survival rate from the risk score was reasonably well correlated with the actual observation of 3-year survival in the external validation data set.

## 4 Discussion

Although EAU guideline on NMIBC appears to be a useful decision-making clinical tool,(Ehdaie et al., 2013) one of the issues in the EORTC risk table is the disproportion in prevalence. In this study, 87.8% of all patients (751 patients) were classified into the intermediate risk group according to EORTC recurrence risk classification in the JT cohort. Xu et al. (2013) and Sakano et al. (2011) showed similar results with 78.0% and 92.5% of patients classified as intermediate risk, respectively. The low frequency of low risk patients could possibly depend in part to the lower rate of G1 tumours in the present study (18.5%) compared with the EORTC trials (43.2%) (Sylvester et al., 2006, Sakano et al., 2011). Because other Asian studies have also reported lower rates of G1 tumours (Sakano et al., 2011, Kikuchi et al., 2009, Hong et al., 2008), there might be racial difference in grade distribution of bladder cancer between Asian and Caucasian populations, similar to the difference between Caucasians and African-Americans (Underwood et al., 2006).

Although some earlier studies reported significant differences in RFS and PFS rates between risk groups (Xu et al., 2013, Altieri et al., 2012, Van Rhijn et al., 2010), other studies including ours (Fig. 1), found that prediction of both recurrence and progression were poorly discriminated by the EORTC tables (Xu et al., 2013, Xylinas et al., 2013, Seo et al., 2010, Fernandez-Gomez et al., 2011, Pillai et al., 2011). Also in another Japanese cohort study (Sakano et al., 2011), no significant differences in the RFS rates were found between low risk and intermediate-low risk groups or between intermediate-high risk and high risk groups. Regarding PFS rates, we could find no significant differences between low risk and intermediate risk (*P* = 0.454) and high-low risk and high-high risk (*P* = 0.338). We stress that our patient population differed significantly from the population analyzed by the EORTC risk group (Sylvester et al., 2006) in terms of geographic location, ethnic background, treatment algorithm and malignant potential. These differences may explain why EORTC table does not work well in Asian populations. These results underline the need for improving current predictive tools (Xylinas et al., 2013) among Asians.

In the EORTC series, only 171 patients (6.5%) were treated with BCG. Subsequently the Spanish Urological Club for Oncological Treatment (CUETO) developed a scoring model that predicted disease recurrence and progression in 1062 patients with NMIBC treated with BCG from four CUETO trials (Fernandez-Gomez et al., 2009). Although both the EORTC risk tables (Sakano et al., 2011, Seo et al., 2010, Fernandez-Gomez et al., 2011, Pillai et al., 2011, Kamat et al., 2007) and the CUETO scoring model (Xylinas et al., 2013) (Pillai et al., 2011) were externally validated and recommended by international guidelines (Babjuk et al., 2013, Burger et al., 2013), (Xylinas et al. (2013), reported that disease recurrence and progression in NMIBC patients were poorly discriminated by both models. At present, the standard adjuvant therapy in patients with NMIBC is the bladder instillation of BCG or chemotherapy. Therefore, it is very important for patients and physician to decide whether or not to receive the adjuvant instillation therapy. The EORTC risk table is, however, of little use for deciding this.

We originally developed the novel risk classification to predict recurrence and progression for Japanese patients with NMIBC to compensate for the shortcomings of the EORTC risk classification. We demonstrated clear and significant differences in RFS rates between the groups (*P* < 0.001; Fig. 3). In addition, unlike in EORTC risk classifications, each risk group had almost equal proportion of patients. This three-tiered risk group stratification made it possible to determine recurrence risk and choose the better adjuvant treatment for individual Japanese patients. In addition, we performed the external validation study to confirm the usefulness of this novel recurrence-risk group stratification in Japanese patients. Even though there were clear differences in clinicopathological backgrounds between the original cohort and the validation set (Table 1), we found an even distribution of patients and significant differences between groups (Fig. 4).

In addition, the scoring items of this novel risk classification system do not include pathological factors such as pathological T classification, concurrent CIS and malignant grade because multivariate analysis showed no significant differences. Therefore, theoretically, we could use this classification before TUR-Bt to predict prognosis. Furthermore, in contrast to the existing risk classifications, this novel risk classification system is characterized by the scoring items including adjuvant bladder instillation therapy of BCG and chemotherapeutic drugs on the first time. At present, the adjuvant therapy for NMIBC is almost BCG instillation or intravesical instillation of chemotherapy and many guidelines recommended these therapies. As a matter of course, this novel classification is not a tool to determine the indication for adjuvant bladder instillation therapy. However, using this novel classification we can evaluate the recurrence-risk classification with or without these adjuvant intravesical instillation therapies.

The limitation of this study is that it was a retrospective analysis. Particularly, our patient cohort included patients treated from 2000 to 2013, which was before immediate post-resection intravesical chemotherapy (Perlis et al., 2013) and maintenance intravesical therapy (Martínez-Piñeiro et al., 2015) were widely-accepted practices in Japan. Immediate intravesical instillation of chemotherapy and BCG maintenance intravesical therapy were performed in just 236 (21.8%) and 33 patients (3.0%), respectively. Also, only 140 (12.9%) patients have had a 2nd TUR performed. Therefore, when BCG and 2nd TUR become widely accepted in Asia as standard therapy, clinical outcome could be different from this present study. Additional factors not included in the EORTC model or in our novel classification such as smoking (Shiels et al., 2014, Burki, 2014), micropapillary histology finding, and the depth of invasion (T1b/c) into lamina propria (Martin-Doyle et al., 2015) could be added to a prognostic model to enhance its usefulness in Asia. Furthermore, in this Japanese study, overall 4.1% patients had disease progression. Compared with 10.7% in EORTC study (Sylvester et al., 2006), this rate is obviously low. In this Japanese cohort, the rate of G1 bladder cancer in JT set (16.8%) and in validation set (12.9%) are clearly lower compared with EORTC study set (43.2%) (Sylvester et al., 2006). Adversely, the rate of G3 bladder cancer in JT set (23.2%) and in validation set (26.8%) are clearly higher compared with EORTC study set (10.4%) (Sylvester et al., 2006). In spite of the high-rate of high-grade NMIBC, in this Japanese study, we can see the very low number of progression compared with Caucasian study. We can't deny the possibility that bladder cancer in Asian people might be of quite different biology in contrast to western cohorts. Therefore, we require the greater consideration the usefulness of our novel classification for Caucasian NMIBC patients.

In conclusion, the number of tumours, tumour size, BCG instillation, and intravesical instillation of chemotherapy were found to be independent predictors for time to recurrence after TUR-Bt in Japanese patients with NMIBC. Our novel, simple, and prognostic classification may not only predict the recurrence risk but greatly help to identify indicators for adjuvant intravesical therapy. Given the fact that comparing with advanced bladder cancer, NMIBC has only a small breakthrough drug (Brower, 2015), further studies with a more patients in a more diversified cohort are required to validate this risk classification and to enhance the effectiveness of existing treatment for Asian patients with bladder cancer.

## Conflict of Interest

None declared.

## Author Contributions

TI and SM analyzed and interpreted data and drafted the initial manuscript. FS, SY, KK, KT, KS, TO, MN, HI, TO, YW, YS, AT, RY, and KN collected data for this study. MT contributed to the data analysis plan and statistical methods used. SH supervised this study. All authors contributed intellectual input to the study design and interpretation of results, and all authors reviewed the manuscript prior to submission. SH approved the final manuscript for submission.

## Figures and Tables

**Table 1:** Patients and tumour characteristics in the JT cohort and the validation set.

		JT set	Validation set
Number of patients		856	641
Age, median (IQR)		71 (64–78)	72 (62–79)
Sex (%)	Male	683 (79.8)	501 (78.2)
	Female	173 (20.2)	140 (21.8)
pT classification (%)	pTa	522 (61.0)	433 (67.6)
	pT1	289 (33.8)	201 (31.3)
	pTis	45 (5.3)	7 (1.1)
Grade (%)	G1	144 (16.8)	83 (12.9)
	G2	513 (59.9)	386 (60.2)
	G3	199 (23.2)	172 (26.8)
Tumour size (%)	< 10 mm	177 (20.7)	90 (14.0)
	≥ 10 mm, < 20 mm	283 (33.1)	251 (39.2)
	≥ 20 mm	396 (46.3)	300 (46.8)
Number of tumours (%)	1	384 (44.9)	370 (57.8)
	2, 3	266 (31.0)	35 (5.5)
	≥ 4	206 (24.1)	236 (36.8)
Concomitant CIS (%)		109 (12.7)	35 (5.5)
2nd TUR-Bt (%)		134 (15.7)	39 (6.1)
BCG induction therapy (%)		220 (25.7)	92 (14.4)
Bladder instillation of chemotherapeutic agents (%)		111 (13.0)	137 (21.4)
EORTC recurrence risk classification (%)			
Low		58 (6.7)	49 (7.6)
Intermediate-low		437 (51.1)	305 (47.6)
Intermediate-high		314 (36.7)	194 (30.2)
High		47 (5.5)	93 (14.5)
EORTC progression risk classification (%)			
Low		191 (22.3)	215 (33.5)
Intermediate		341 (39.8)	216 (33.7)
High-low		243 (28.4)	154 (24.0)
High-high		81 (9.5)	56 (8.7)
Follow up period, median (IQR)		31 (15–48)	43 (17–73)

**Table 2:** Univariate and multivariate RFS rates according to clinicopathological risk factors.

Risk factor		Univariate			Multivariate			Risk score
		Risk ratio	98% CI	*P*	Risk ratio	98% CI	*P*	
Age (< 70 vs.)	≥ 71	1.05	0.85–1.29	0.671	0.96	0.78–1.19	0.720	
Sex (female vs.)	Male	1.10	0.83–1.44	0.524	1.07	0.93–1.42	0.622	
Number of tumours (simple vs.)	2,3	1.19	0.93–1.53	0.160	1.46	1.14–1.89	0.003	8
	≥ 4	1.39	1.06–1.81	0.016	1.93	1.46–2.55	< 0.001	13
Tumour size (< 1 cm vs.)	≥ 1 cm, < 2 cm	1.28	0.93–1.79	0.132	1.24	0.89–1.76	0.204	4
	≥ 2 cm	1.74	1.30–2.39	< 0.001	1.77	1.30–2.47	< 0.001	11
T stage (Ta vs.)	T1	1.12	0.89–1.39	0.333	0.93	0.72–1.18	0.527	
Concurrent CIS (no vs.)	Yes	0.63	0.42–0.89	0.010	1.06	0.68–1.61	0.796	
Grade (G1 vs.)	G 2–3	1.11	0.84–1.48	0.470	1.03	0.77–1.40	0.840	
BCG (yes vs.)	No	2.38	1.80–3.22	< 0.001	3.13	2.27–4.40	< 0.001	22
Chemotherapy (yes vs.)	No	1.36	0.99–1.92	0.058	1.67	1.21–2.36	0.001	11

**Table 3:** 3-year recurrence probability in the JT set.

Total score	3-year recurrence probability
0	8.4%
5	10.7%
10	13.5%
15	17.0%
20	21.3%
25	26.5%
30	32.6%
35	39.8%
40	47.9%
45	56.7%
50	65.8%
55	74.8%
60	83.0%
